# Depression detection through dual-stream modeling with large language models: a fusion-based transfer learning framework integrating BERT and T5 representations

**DOI:** 10.3389/fdata.2025.1651290

**Published:** 2026-02-04

**Authors:** Na Wang, Weijia Zhang, Raja Kamil, Ian Renner, Syed Abdul Rahman Al-Haddad, Normala Ibrahim, Zhen Zhao

**Affiliations:** 1 Faculty of Engineering, Universiti Putra Malaysia, UPM Serdang, Serdang, Selangor, Malaysia; 2 School of Information and Physical Sciences, The University of Newcastle, Callaghan, NSW, Australia; 3 School of Automation, Guangdong Polytechnic Normal University, Guangzhou, Guangdong, China; 4 Faculty of Medicine and Health Sciences, Universiti Putra Malaysia, UPM Selangor, Selangor, Malaysia; 5 Department of Electrical Engineering, Faculty of Engineering, Universiti Malaya, Kuala Lumpur, Malaysia

**Keywords:** 1DCNN, BERT, depression, E-DAIC, T5, text, transfer learning, transformer

## Abstract

Millions of people around the world suffer from depression. While early diagnosis is essential for timely intervention, it remains a significant challenge due to limited access to clinically diagnosed data and privacy restrictions on mental health records. These limitations hinder the training of robust AI models for depression detection. To tackle this, this article proposes a parallel transfer learning framework for depression detection that integrates BERT and T5 through a fusion mechanism, combining the complementary advantages of these two large language models (LLMs). By integrating their semantic embeddings, the method captures a broader range of linguistic cues from transcribed speech. These embeddings are processed through a model with two parallel branches: a one-dimensional convolutional neural network and a dense neural network are used to construct each branch for preliminary prediction, which are then fused for final prediction. Evaluations on the E-DAIC dataset demonstrate that the proposed method outperforms baseline models, achieving a 3.0% increase in accuracy (91.3%), a 6.9% increase in precision (95.2%), and a 1.7% improvement in F1-score (90.0%). The experimental results verify the effectiveness of BERT and T5 fusion in enhancing depression detection performance and highlight the potential of transfer learning for scalable and privacy-conscious mental health applications.

## 1 Introduction

Depression is also referred to as major depressive disorder (MDD). It is a widespread mental illness that impacts a significant percentage of people globally. According to the statistics of the World Health Organization, approximately 332 million people in the world have depression (Herrman et al., 2019). Depression is characterized by symptoms such as persistent sadness, loss of interest in daily activities, irritability, hopelessness, changes in appetite or weight, and low self-worth. In more severe cases, it may also result in suicidal thoughts or actions.

Diagnosing depression is inherently complex, typically requiring the expertise of trained mental health professionals. While early detection is essential for effective intervention, access to timely diagnosis remains limited due to high costs and inadequate access to mental health care, particularly in rural and underserved areas (Semrau et al., 2019). Artificial intelligence (A.I.) has shown promise in recent years in various medical applications (Uppal et al., 2023; Zafar et al., 2023; Wang et al., 2025; Zhao et al., 2023), including mental health assessment. However, the development of AI-based depression detection systems is hindered by several challenges, chief among them being the shortage of clinically validated data for model training.

Most datasets that are accessible to the general public for depression detection are relatively small and exhibit severe class imbalance. For instance, the Extended Distress Analysis Interview Corpus (E-DAIC) includes only 275 participants, of whom just 66 (24%) are diagnosed with MDD. Similarly, the Chinese Multimodal Depression Corpus (CMDC) and the Multimodal Open Dataset for Mental-health Analytics (MODMA) contain merely 78 and 52 participants, respectively, with fewer than half representing depressed individuals. The potential of AI models to generalize across various populations and therapeutic scenarios is severely limited by these dataset size and representativeness constraints.

Among various methods of detecting depression, the investigation of linguistic features in patient speech has been regarded as especially beneficial. Text features offer a more abundant level of interpretability compared to auditory or visual modalities. Linguistic markers, particularly those with absolutist terms such as “NEVER” indicated by phrases such as “I NEVER want to wake up” (Adam-Troian et al., 2022), and desperation or fatigue expressions, more commonly represented by “I am so tired all the time,” are found to correlate with depressive symptoms. Conventional clinical instruments, including the Beck Depression Inventory (Beck et al., 1996), Self-Rating Depression Scale (Zung, 1965), and Patient Health Questionnaire (PHQ; Spitzer et al., 1999), rely strongly on patient feedback in verbal or written format, thereby highlighting the central role that textual information plays in depression evaluation.

In spite of these strengths, the variation in patients' language abilities, emotional expressions, and cultural contexts poses significant obstacles to the construction of representative textual datasets. Without sufficient diversity and sample populations, artificial intelligence systems are unable to successfully pick up on subtle cues of indicators of depression and might not be as generalizable to real-life situations. It is necessary to overcome these limitations to fully harness the potential of artificial intelligence-based depression intervention systems.

To resolve limitations related to insufficient training data, we recommend using a transfer learning approach based upon deep pre-trained language models to strengthen performance in minimal, clinically annotated datasets. Specifically, our model utilizes two big language models (LLMs) to take advantage of their pre-trained knowledge and two different neural network designs, namely a one-dimensional convolutional neural network (1DCNN) and a fully connected neural network typified by dense layers combined with a dropout mechanism for automatic depression detection. By fine-tuning the model using diagnostic labels and harnessing the linguistic knowledge encoded in the LLMs, the proposed architecture enhances the system's ability to identify depressive symptoms from textual input.

The following is a summary of this research's main contributions.

1. Introducing a dual-stream transfer learning fusion framework that leverages the complementary strengths of two pre-trained large language models (LLMs), BERT, and T5, combined with 1D convolutional and dense neural networks, enabling robust depression detection by capturing diverse linguistic representations.
2. Designing a lightweight logical “AND fusion” strategy for integrating the outputs of all four branches as a conservative agreement based decision rule, with the aim of enhancing prediction reliability and precision rather than introducing a novel ensemble mechanism.
3. Conducting comprehensive ablation studies to evaluate the individual and combined contributions of BERT and T5 embeddings, as well as different architectural variants, providing deeper insights into their effectiveness for clinical depression detection.
4. Benchmarking the proposed method against common machine learning models (including traditional machine learning models and deep learning models), as well as the state-of-the-art studies on the E-DAIC dataset. The findings of this study offer a reference for future study on the application of LLMs in related domains.

This paper's remaining sections are arranged as follows: Section 2 provides an overview of previous studies pertinent to this research; Section 3 describes the workflow, dataset, data processing steps, and model structure; Section 4 outlines the experiment and evaluation setups; Section 5 presents the experiment configurations and evaluation criteria; and finally, Section 6 concludes the study with final remarks.

## 2 Related work

Speech text provides valuable insights for depression evaluation, which motivates researchers to develop automatic depression detection methods using textual data. Among the deep learning approaches, 1DCNN and variants of Recurrent Neural Networks (RNNs), such as Long Short-Term Memory (LSTM) and BiLSTM, have been widely employed for these tasks.

Convolutional neural networks, initially proposed for image processing (LeCun et al., 1989), were utilized for one-dimensional data in the mid-2010s, tackling difficulties in natural language processing, time-series analysis, and signal processing. An early milestone in this direction was the work of Kim et al., who applied 1DCNNs to text classification in 2014 (Kim, 2014).

Complementing CNNs, Long Short-Term Memory (LSTM) networks, proposed by Hochreiter and Schmidhuber (1997), addressed the vanishing gradient issue of traditional RNNs, allowing the effective learning of long- and short-term correlations. Building on LSTM, Graves and Schmidhuber (2005) proposed BiLSTM, which combines forward and backward LSTMs to capture bidirectional dependencies, making it suitable for sequential data processing tasks, e.g., speech recognition, automatic translation, and classifications.

In 2023, Wani et al. explored depression screening using Word2Vec (Church, 2017) and TF-IDF (Baena-García et al., 2011) features combined with CNN and LSTM models (Ahmad Wani et al., 2023). They achieved accuracies of 99.01% with Word2Vec (CNN + LSTM) on data sourced from Facebook, Twitter, and YouTube. However, one of the study's limitations is its dependence on non-clinically diagnosed data, raising concerns about its applicability in clinical settings.

To address limitations in data and feature extraction, transfer learning techniques have emerged as a promising approach across various research domains and have shown effectiveness in a range of language-related applications.

The BERT model (Vaswani et al., 2017), pre-trained on massive corpora such as BooksCorpus (Zhu et al., 2015) and English Wikipedia, has become an essential building block of transfer learning techniques. Such proficiency in learning knowledge from long and uninterrupted texts through document-level training has been key to its applicability in tasks that need deep contextual understanding. For instance, Milintsevich et al. (2023) applied the RoBERTa (Liu et al., 2019) model, an adaptation of BERT, for depression prediction based on clinical transcripts from the DAIC dataset. According to their study, depression detection revealed a macro-F1 score of 73.9 within a binary setting, citing the need for clinically validated datasets.

Zhang and Guo (2024) presented the MDSD-FGPL algorithm, integrating BERT and T5 encoders in fine-grained prompt learning, through a research project carried out in 2024. The multi-tier detection approach yielded an F1-score of 0.8276 for binary classification, hence pointing out the advantages of encoder integration. Hadikhah Mozhdehi and Eftekhari Moghadam (2023) applied Emotional BERT toward tasks of emotion recognition on the Wang and MELD datasets through another investigation, further proving the effectiveness of transfer learning in deriving contextual knowledge from minimal datasets.

The T5 model, described by Raffel et al. (2020), has been widely applied to a variety of text-centered analytical tasks (Bao et al., 2024). The model's flexibility is further demonstrated by the Sensory-T5 version presented by Zhao et al. (2025), which incorporates sensory information to enhance emotion classification accuracy. This methodology has led to significant precision and F1 score improvement on various datasets.

Inspired by a similar effort, Lu et al. (2025) also fine-tuned T5 for their ABSA and achieved remarkable improvement through their application of data augmentation techniques and implicit rationale-driven information management. Chawla et al. (2024) also identified emotional dimensions that influence the negotiation process, particularly with regard to outcome predictions such as satisfaction and perceptions of partners. Incorporating the CaSiNo dataset with emoticons, Linguistic Inquiry and Word Count, and T5 models, they conducted a comparison whereby T5-Reddit performed better than other models in detecting subtle emotional expressions.

As impressive as the breakthroughs achieved through leveraging pre-trained models have been for various text analytical applications, most available works tend to be based upon a single model developed for a specific architecture or a single configuration of tasks. Even though these models have delivered impressive performances, overreliance upon a solitary model has the potential to compromise generalizability, especially when applied in cases like detecting clinical depression, where access to annotated knowledge is sparse and contextual centrality matters.

Considering these limitations, the current work proposes initiating an innovative dual-language model integration. A methodological strategy that combines benefits of two sizable pre-trained language models: BERT and T5. The models have complementary characteristics in structural design and learning methods. BERT is an encoder-based model that is particularly designed to handle bidirectional contexts. The use of contextual features in a sentence reinforces its ability to capture subtle semantic relationships and complex syntactic relationships. On the other hand, T5 is a text-to-text transformer model based on a combined encoder-decoder model that is ideally suited to generate and reconstruct text content, thus demonstrating a better ability to generalize and abstract across diverse tasks. By combining the models, the current approach leverages the accuracy of BERT in producing contextual embeddings (Devlin et al., 2019) and the versatility of T5 in capturing task-agnostic patterns through its unified text-to-text framework (Raffel et al., 2020), ultimately leading to more accurate and efficient detection of depression from transcribed speech.

## 3 Methodology

**Figure 1:**
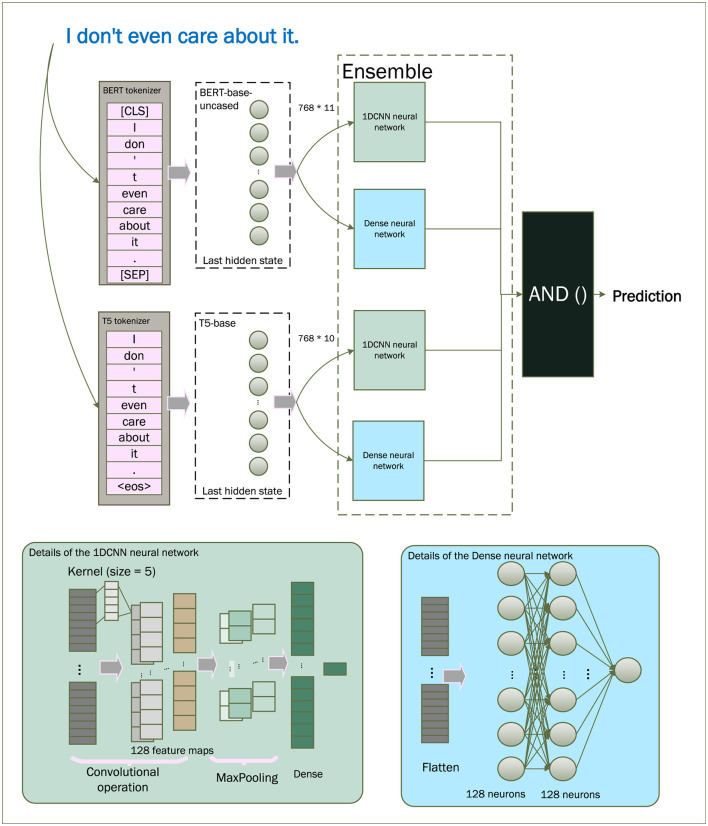
The workflow of the proposed method.

The initial step involves pre-processing raw text transcripts, which is integrated into the proposed framework. As illustrated in Figure 1, the framework takes advantage of the collaborative strengths of transformer-based pre-trained large language models, specifically BERT and T5, both of which are highly reputed for their efficiencies in natural language processing. These models possess the capability to learn subtle linguistic patterns, grammatical structures, and other language attributes (Rodríguez-Ibánez et al., 2023), and are assumed to capture transferable knowledge.

To effectively utilize this pre-trained knowledge for our task, both models are fine-tuned using pre-processed data tailored to depression prediction. The processed text is fed into the fine-tuned BERT and T5 encoders, generating two distinct sets of embeddings. These embeddings are subsequently passed through parallel 1DCNN and Dense branches to extract task-relevant features and produce preliminary predictions.

The resulting predictions are then passed to a fusion module that performs a logical “AND” operation for the final output.

### 3.1 Dataset and pre-processing

Clinically diagnosed datasets are scarce, as most publicly available datasets are derived from social media posts where labels are self-claimed. While these datasets offer valuable insights, they lack the reliability of clinically confirmed ground truth. Some datasets are built from samples collected from clinically diagnosed MDD patients; however, the majority of these are not publicly accessible. In contrast, the E-DAIC dataset is not only clinically reliable but also publicly available, with baseline models provided to enable fair comparisons of model performance.

The E-DAIC dataset, developed from clinical interviews, is designed to aid in diagnosing psychological disorders including anxiety, depression, and post-traumatic stress disorder (PTSD). It includes recordings of the conversations between the interviewers and the research subjects, along with transcripts capturing both parties' spoken words, providing a rich linguistic context for analysis. Compared to the DAIC dataset, E-DAIC offers a larger sample size, which enhances its utility for research.

The dataset includes recordings from 275 subjects. 66 (35 males, 31 females) of whom were diagnosed with depression at the time of recording, while 209 (135 males, 74 females) were categorized as health controls (HCs). PHQ-8 scores of each subject are provided. A subset of the sessions in the E-DAIC dataset was collected semi-automatically, with a virtual interviewer controlled by a human. The rest were gathered using an AI-controlled agent. It uses automated modules for perception and behavior generation to function completely independently. The subjects comprised U.S. military veterans and recruits from the general public in the Los Angeles area.

Detailed summaries of the data collection methods and participant demographics are provided in Tables 1, 2.

**Table 1:** Dataset information of the E-DAIC.

**Attribute**	**Details**
Disorders	Depression, PTSD
Diagnosis basis	PHQ-8, PCL-C
Number of participants	275
Data modalities	Visual, Audio, Text

PCL-C, PTSD Checklist - Civilian Version.

**Table 2:** Gender and MDD proportion of the E-DAIC dataset.

**Gender**	**HC**	**MDD**	**Positive ratio**
Male	135	35	20.6%
Female	74	31	29.5%
Total	209	66	24.0%

The following preprocessing steps were implemented for preprocessing:

- Trimming irrelevant sections: the interview scripts' initial 90 s and last 40 s were eliminated. These portions predominantly contained answers to introductory remarks, general questions (e.g., “Where are you from?”), greetings, and goodbyes, which were deemed irrelevant for the analysis.
- Only participant utterances were retained for textual analysis, while interviewer speech was excluded.
- Neutral sentence removal: sentences consisting of one neutral word only, e.g., “yes” or “ok,” were excluded, as they lacked meaningful information for classification.
- Labeling: the scripts were assigned labels based on PHQ-8 binary scores, with “1” indicating MDD and “0” indicating HC.
- Balancing classes: to achieve an equal distribution between MDD and HC samples and prevent model bias during training, we adopted a random undersampling strategy. Among the available options, we chose undersampling to preserve the full set of clinically diagnosed MDD cases. Ensuring that all MDD samples originate from genuine, clinically diagnosed patients is crucial in medically sensitive contexts such as ours (Fernández et al., 2015). In contrast, upsampling methods typically introduce synthetic data, which may compromise the authenticity and clinical reliability of the dataset.

Table 3 provides examples of the preprocessed text and labels.

**Table 3:** Examples of the pre-processed text data.

**Text transcript**	**Label**
No, it's just too rough trying to pick up all the pieces	1
Sleeping all the time, eating too much, arguing, screaming at this	1
I just couldn't find work, and so I had to settle for doing that for right now	1
I applied from anywhere and everywhere	1
My parents just buried their daughter six months ago; they don't want to bury their other daughter	1
I play sports: volleyball, softball, biking, walking	0
I'm a little more rigid than most people, but it was okay, not bad	0
Maybe more disciplined. I can take orders very well. I'm not afraid of most situations	0
He knows exactly what I'm going through, and he's one hundred percent behind me, and I love him very much	0
Yeah, I mean they've always gave me great advice	0

The E-DAIC dataset was selected for the current study since it is clinically trustworthy and publicly accessible. However, a small dataset sample size makes it hard to train deep learning models from the beginning. In tackling such a weakness, transfer learning was used to improve not just performance but also generalization ability. Leveraging knowledge embedded in pre-trained models, transfer learning considerably expands exploration of the E-DAIC dataset. Transfer learning is leveraged in the current project by fine-tuning transformer-based LLMs: BERT and T5.

### 3.2 BERT and T5

Both BERT and T5 are pre-trained large language models (LLMs) based on the Transformer architecture. They act as contextual feature extractors for input text, generating dense vector representations. Table 4 summarizes the architectural and training differences between the original Transformer, B-base (BERT-base), and T-base (T5-base).

**Table 4:** Key differences between transformer, BERT, and T5.

**Aspect**	**Transformer base**	**B base (BERT)**	**T base (T5)**
Architecture	Encoder-Decoder	Encoder-only	Encoder-Decoder
Contextualization	Unidirectional encoder and decoder	Bidirectional encoder	Bidirectional encoder, Unidirectional decoder
Pre-training objective	None (original)	MLM + NSP	Span Corruption
Model size (parameters)	65M	110M	220M

Let the input sequence be represented as:


$$\begin{array}{l} X=\left\{x_{1},x_{2},\ldots ,x_{N}\right\},\;x_{i}\in \text{𝕋}, \end{array}$$ (1)

where 𝕋 denotes the token space. The contextual embeddings extracted by each model are denoted by:


$$\begin{array}{l} {\text{E}}^{B}=B\left(X\right),\;\text{BERT embeddings}, \end{array}$$ (2)

$$\begin{array}{l} {\text{E}}^{T}=T\left(X\right),\;\text{T5 embeddings}, \end{array}$$ (3)

where


$$\begin{array}{l} {\text{E}}^{B}=\left\{{\text{h}}_{1}^{B},\ldots ,{\text{h}}_{N}^{B}\right\},\;{\text{h}}_{i}^{B}\in {\mathbb{R}}^{d}, \end{array}$$ (4)

$$\begin{array}{l} {\text{E}}^{T}=\left\{{\text{h}}_{1}^{T},\ldots ,{\text{h}}_{N}^{T}\right\},\;{\text{h}}_{i}^{T}\in {\mathbb{R}}^{d}. \end{array}$$ (5)

To obtain fixed-size embeddings, we apply mean pooling across each token sequence:


$$\begin{array}{l} {\bar{\text{e}}}^{B}=\frac{1}{N}\overset{N}{\underset{i=1}{\sum }}{\text{h}}_{i}^{B}, \end{array}$$ (6)

$$\begin{array}{l} {\bar{\text{e}}}^{T}=\frac{1}{N}\overset{N}{\underset{i=1}{\sum }}{\text{h}}_{i}^{T}, \end{array}$$ (7)

where ${\bar{\text{e}}}^{B}\in {\mathbb{R}}^{d}$ and ${\bar{\text{e}}}^{T}\in {\mathbb{R}}^{d}$ denote the sentence-level embeddings for the input *X* from BERT and T5, respectively.

These vectors are saved in NumPy format (.npy) and serve as inputs to downstream classifiers. The embedding extraction process is summarized in Algorithm 1.

Algorithm 1 **Embedding generation using pre-trained LLMs.**

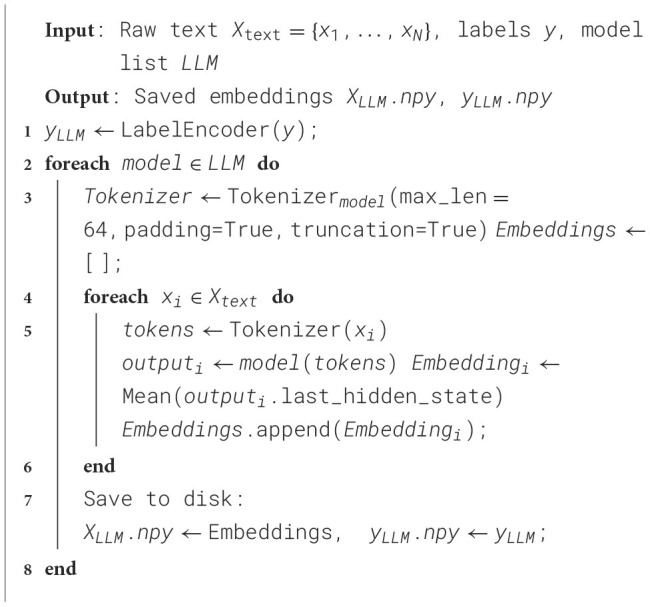

### 3.3 Classification and fusion

To classify the embeddings, we use two neural network classifiers:

- *M*_*C*_: a 1D Convolutional Neural Network (1DCNN),
- *M*_*D*_: a fully connected Dense Neural Network (DNN).

The 1DCNN *M*_*C*_ operates on the input vector *x* ∈ ℝ^*d*^ with kernel *w* ∈ ℝ^*m*^, producing an output *y*_*i*_ at position *i* as:


$$\begin{array}{l} y_{i}=\overset{m}{\underset{j=1}{\sum }}x_{i+j-1}\cdot w_{j}. \end{array}$$ (8)

Each model computes a probability score indicating the likelihood of depression.


$$\begin{array}{l} p_{1}=M_{C}\left({\bar{\text{e}}}^{B}\right),\;p_{2}=M_{D}\left({\bar{\text{e}}}^{B}\right), \end{array}$$ (9)

$$\begin{array}{l} p_{3}=M_{C}\left({\bar{\text{e}}}^{T}\right),\;p_{4}=M_{D}\left({\bar{\text{e}}}^{T}\right). \end{array}$$ (10)

A final prediction is made using a conservative logical AND fusion:


$$\begin{array}{l} {ŷ}_{\text{final}}=𝕀\left[\left(p_{1}>\tau \right)\wedge \left(p_{2}>\tau \right)\wedge \left(p_{3}>\tau \right)\wedge \left(p_{4}>\tau \right)\right], \end{array}$$ (11)

where τ ∈ [0, 1] is the decision threshold (defaultτ = 0.5), and *I*[·] is the indicator function returning 1 if the condition is true. A sample is labeled as MDD only if all four models agree.

The classification and fusion procedure is summarized in Algorithm 2.

Algorithm 2 **Neural network fusion**

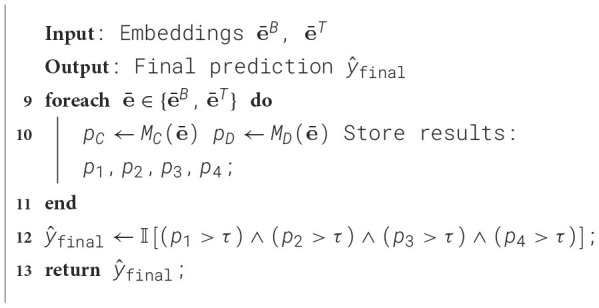

## 4 Experiment configurations and evaluation criteria

The experiment's hardware is a workstation with an AMD Ryzen 9 CPU and 64 GB RAM. The software environment is the Windows 11 operating system, Jupyter IDE, and Python 3.11. The models were trained using a CPU.

The experimental results are assessed using the following metrics: accuracy, precision, recall, and F1 score. These metrics offer a thorough comprehension of the model's functionality. The equations for these metrics are defined as follows:


$$\begin{array}{l} \text{Accuracy}=\frac{\text{TP}+\text{TN}}{\text{TP}+\text{FP}+\text{TN}+\text{FN}}, \end{array}$$ (12)

$$\begin{array}{l} \text{Precision}=\frac{\text{TP}}{\text{TP}+\text{FP}}, \end{array}$$ (13)

$$\begin{array}{l} \text{Recall}=\frac{\text{TP}}{\text{TP}+\text{FN}}, \end{array}$$ (14)

$$\begin{array}{l} \text{F1}=\frac{2\text{precision}\times \text{recall}}{\text{precision}+\text{recall}}, \end{array}$$ (15)

where TP, TN, FP, and FN represent the counts of true positives, true negatives, false positives, and false negatives in the predictions, respectively. Additionally, the study involves plotting the Receiver Operating Characteristic (ROC) curves for the learning models for comparison purposes. The ROC curve depicts the true positive rate (TPR) vs. the false positive rate (FPR) at various threshold settings. Furthermore, the area under the curve (AUC) was calculated. An increased AUC signifies improved overall performance.

## 5 Experimental results

In this section, we examine and compare the traditional text feature extraction algorithms and transfer learning-based text embeddings using both conventional ML and deep learning models. The study evaluates six conventional machine learning models–Logistic Regression (LR), Support Vector Machine (SVM), Random Forest (RF), Naive Bayes (NB), K-Nearest Neighbor (KNN), and Decision Tree (DT)–alongside five deep learning models designed for one-dimensional text data analysis. The deep learning models include a fully connected dense neural network, a 1DCNN, a recurrent neural network (RNN), a gated recurrent unit (GRU), an LSTM, and a bidirectional long short-term memory network (BiLSTM).

### 5.1 Experiments of traditional text feature extraction algorithms

To evaluate transfer learning's efficacy in improving models' performance in this task, we first adopted the commonly used traditional text feature extraction algorithms, namely term frequency-inverse document frequency (TF-IDF), Keras frequency-based tokenizer (KFT), Bag of Words (BoW), and N-grams on both conventional MLs and deep learning models.

**Figure 2:**
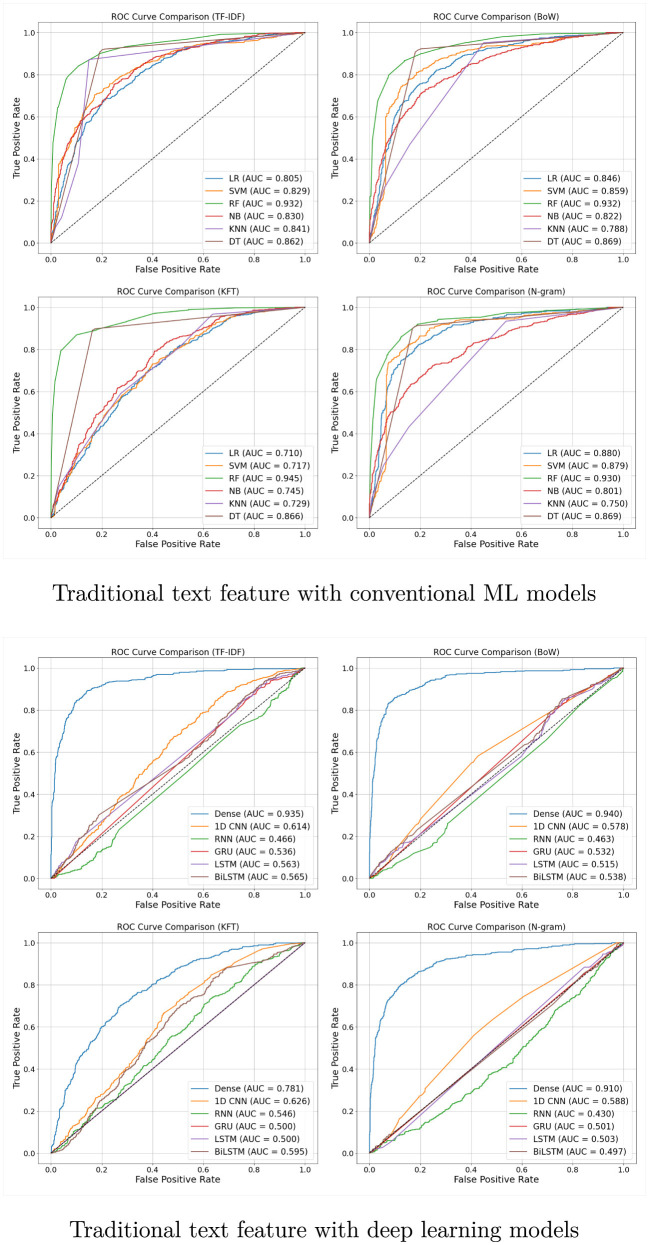
ROC of models using traditional text feature extraction algorithms. The upper panel shows results from conventional machine learning models, while the lower panel shows results from deep learning models.

Figure 2 presents the ROC curves generated using traditional text features for both conventional ML models and deep learning models. Among the conventional ML models, RF and DT demonstrated superior performance, with RF achieving the highest AUC of 94.5% when using KFT features. In contrast, the deep learning models generally underperformed, with the notable exception of the fully connected Dense Neural Network, which achieved an AUC of 94.0% using BoW features.

The RF model excels with a higher true positive rate (TPR) at lower false positive rates (FPR), reflecting its efficiency in correctly identifying positive samples. This balance between sensitivity and specificity underscores its effectiveness as a classifier. Furthermore, RF consistently achieves the highest AUC across various feature extraction methods, demonstrating its robustness to diverse data representations and its superior ability to distinguish between classes.

The deep learning models, with the exception of dense neural networks constructed solely with dense layers, showed poor performance when tested with traditional text feature extraction algorithms. The results of the other deep learning models are not reported, as their poor performance suggests that combining traditional text feature extraction algorithms with these models is not an effective approach for this task. Among the tested models, the dense neural network using BoW achieved an accuracy of 85.9%, outperforming all other deep learning models.

Table 5 reports the accuracy, precision, recall, and F1-score of the learning models using traditional text feature extraction algorithms. As shown, the highest accuracy was achieved by RF using TF-IDF, with an accuracy of 88.3%. This method (TF-IDF + RF) is selected as the baseline for evaluating the effectiveness of transfer learning. Overall, the results indicate that conventional ML models outperform deep learning models when traditional extraction algorithms are applied.

**Table 5:** The performance of learning models with traditional text feature algorithms (Non-TL).

**Model**	**Feature**	**Accuracy (%)**	**Precision (%)**	**Recall (%)**	**F1 (%)**
LR	TF-IDF	73.0	72.8	72.7	72.7
	KFT	65.1	64.9	64.6	64.6
	BoW	77.9	77.9	77.5	77.6
	N-gram	**81.0**	**80.9**	**80.8**	**80.8**
SVM	TF-IDF	75.7	75.7	75.8	75.6
	KFT	64.9	64.7	64.6	64.7
	BoW	81.2	81.2	81.0	81.0
	N-gram	**83.5**	**83.4**	**83.3**	**83.4**
RF	TF-IDF	**88.3** ^*^	**88.3** ^*^	**88.3** ^*^	**88.3** ^*^
	KFT	86.8	86.8	86.8	86.8
	BoW	86.1	86.1	86.1	86.1
	N-gram	86.7	86.6	86.7	86.6
NB	TF-IDF	**74.6**	**74.6**	**74.7**	**74.5**
	KFT	59.2	65.0	56.3	50.5
	BoW	74.1	74.1	74.3	74.1
	N-gram	73.7	73.5	73.5	73.5
KNN	TF-IDF	57.1	63.6	53.9	45.6
	KFT	66.3	66.1	**65.8**	**65.8**
	BoW	**66.9**	**68.2**	65.4	64.9
	N-gram	65.4	66.9	63.8	62.9
DT	TF-IDF	85.3	85.6	85.7	85.3
	KFT	86.1	86.1	86.4	86.1
	BoW	86.0	86.1	86.3	85.9
	N-gram	**86.7**	**86.7**	**86.7**	**86.5**
Dense	TF-IDF	85.2	85.4	85.3	85.2
	KFT	67.0	70.1	67.6	67.0
	BoW	**85.9**	**86.0**	**85.9**	**85.9**
	N-gram	85.5	85.5	85.5	85.5

For each model, the highest scores are highlighted in bold.

^*^denotes the highest performance across all learning models and text features.

### 5.2 Experiments of transfer learning methods

Next, we investigate the effect of transfer learning by adopting two pre-trained models: BERT and T5. BERT represents an encoder-only model optimized for bidirectional contextual understanding, while T5 follows a sequence-to-sequence, text-to-text pre-training paradigm based on span corruption. Including both models allows us to examine whether generative-style pre-training yields complementary representations for depression-related language beyond encoder-only architectures. For a fair comparison, the embeddings generated from these BERT and T5 were used to train the same conventional ML and deep learning models.

In Table 6, we observe that the Dense model performs best across most metrics when using BERT embeddings, while the 1DCNN outperforms all models with T5 embeddings, achieving the highest accuracy, F1-score, and AUC. Overall, the 1DCNN emerges as the most robust model, excelling particularly with T5 embeddings, while the dense layer consistently delivers strong performance with both embeddings, showing a slight edge with BERT. In contrast, RNN, LSTM, and BiLSTM models consistently underperform compared to other models regardless of the embedding type.

**Table 6:** Comparison of the models using transfer learning.

**Model**	**Embedding**	**Accuracy (%)**	**Precision (%)**	**Recall (%)**	**F1 (%)**
LR	BERT	69.04	66.31	67.03	66.67
	T5	**72.47**↓	**70.01**↓	**70.68**↓	**70.34**↓
SVM	BERT	69.79	67.25	67.43	67.34
	T5	**71.29**↓	**68.87**↓	**69.05**↓	**68.96**↓
RF	BERT	86.10	84.25	86.76	85.49
	T5	**87.27**↓	**85.17**↓	**87.70**↓	**86.42**↓
NB	BERT	61.55	57.23	66.35	61.45
	T5	**63.17**↓	**58.87**↓	**67.30**↓	**62.80**↓
KNN	BERT	**70.66**	**68.24**	68.24	**68.24**
	T5	69.41	66.32	**68.65**	67.46
DT	BERT	**86.83**↑	**82.53**↓	**90.68**↑	**86.41**↓
	T5	85.58	80.99	89.86	85.20
Dense	BERT	**87.33**↑	**84.38**↓	**89.05** ↑	**86.65**↑
	T5	86.14	83.29	87.57	85.38
1DCNN	BERT	**89.45** ^*^	**88.02** ^*^	**89.32**	**88.67** ^*^
	T5	88.45	86.56	88.78	87.66
RNN	BERT	**85.46**	80.88	**89.73**	**85.07**
	T5	77.65	**82.15**	65.95	73.16
GRU	BERT	**85.21**	78.74	**93.11** ^*^	**85.33**
	T5	85.02	**81.65**	87.16	84.31
LSTM	BERT	67.04	64.64	**63.24**	**63.93**
	T5	**67.29**	**66.12**	59.86	62.84
BiLSTM	BERT	52.25	48.33	48.78	48.55
	T5	**72.97**	**67.79**	**79.05**	**72.99**

The highest value for each model is highlighted in bold.

Arrows (↑/↓) indicate the increase or decrease compared to the highest score of the corresponding non-TL method for the same learning model. ^*^denotes the highest performance across all learning models using TL embeddings.

Traditional machine learning methods are routinely outperformed by deep learning models, especially 1DCNN and dense. 1DCNN (BERT) achieves the highest accuracy (89.45%) and F1-score (88.67%), highlighting its robustness in this task. Among traditional ML models, RF and DT perform better than the other models but do not show an increase compared to non-transfer learning models. While GRU (BERT) excels in recall (93.11%), minimizing false negatives, models like RNN and LSTM show moderate performance. Simpler models such as Naive Bayes (NB) exhibit significant drops in F1-score.

The comparison between non-TL and TL models reveals distinct performance patterns. Traditional models such as RF and DT show competitive results in the non-TL setting, particularly when combined with TF-IDF features, with RF achieving the highest scores in accuracy, precision, recall, and F1-score. This suggests these models are well-suited to conventional feature representations.

In contrast, TL models using BERT and T5 embeddings consistently outperform non-TL approaches. Among them, 1DCNN with BERT embeddings achieves the highest accuracy (89.45%) and F1-score (88.67%), surpassing the best non-TL setup (RF + TF-IDF: 88.3%). This highlights the benefit of leveraging contextual embeddings for improved classification accuracy.

**Figure 3:**
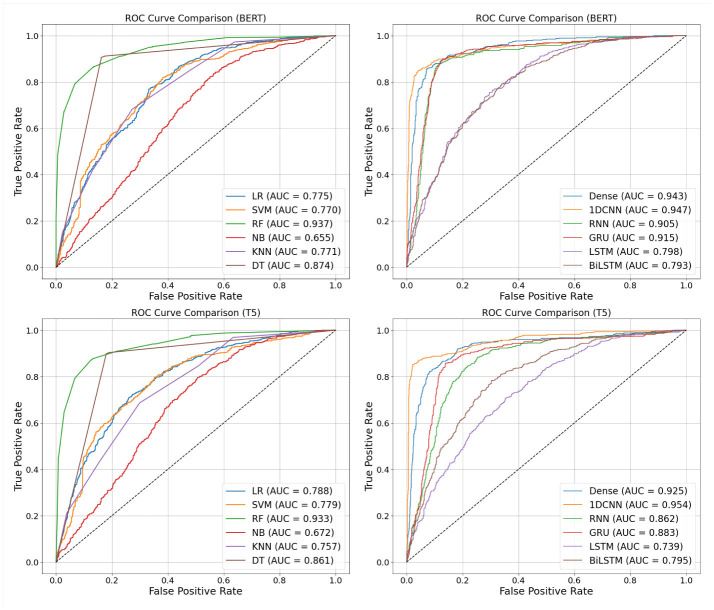
Performance of transfer learning using traditional machine learning models and deep learning models.

As illustrated in Figure 3, TL-based models produce ROC curves that shift further left, corresponding to higher AUC scores and improved true positive rates, especially at low false positive rates. This behavior underscores the increased sensitivity and robustness of transfer learning in text-based depression detection.

Given the effectiveness of 1DCNN and dense models using BERT and T5 embeddings in the above experiments, these models emerged as promising candidates for developing a superior approach to this task. As a result, we selected them for further experiments.

**Figure 4:**
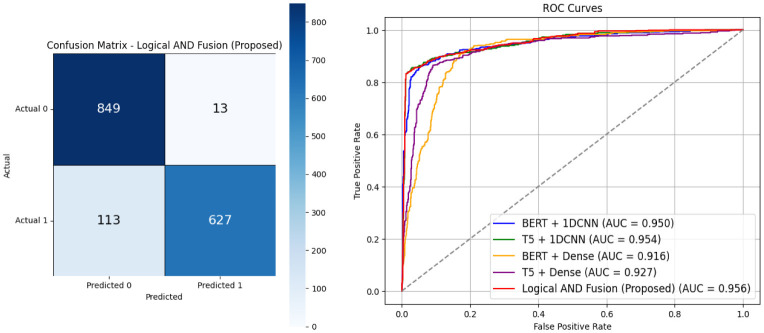
The confusion matrix and ROC of the proposed dual-stream transfer learning.

As illustrated in Figure 4, the confusion matrix of the proposed dual-stream model demonstrates relatively balanced performance across both classes. Notably, the number of false negatives (113) is substantially lower than that of false positives (13), indicating that the model prioritizes sensitivity. This is particularly advantageous in early-stage depression screening, where maximizing recall is often more critical than specificity.

In terms of ROC performance, the fusion approach–integrating predictions from four branches (BERT + Dense, BERT + 1DCNN, T5 + Dense, and T5 + 1DCNN)–achieved the highest AUC of 0.956, as shown in Figure 4, outperforming each individual model. By shifting the ROC curve further toward the top-left corner, the fusion method yields a higher true positive rate at a lower false positive rate.

### 5.3 Ablation study

As observed, integrating BERT and T5 embeddings with 1DCNN and Dense architectures demonstrates strong discriminative ability, achieving high scores across all evaluation metrics and highlighting their potential as a promising approach. To further optimize the architecture, an ablation study was carried out to identify the best structure for the task.

During training BERT and T5 for predictive tasks, baseline models were created to test the effectiveness of embeddings without considering architectural bias. BERT achieved impressive performance, recording an accuracy rate of 88.8%, an F1-score of 88.0%, and an AUC of 92.8%. Its impressive recall rate of 89.5% indicates that it has good proficiency in exactly categorizing positive examples, making it very apt for operations that require minimizing false negatives. On the contrary, T5 had much lower performance scores, recording an accuracy rate of 64.1%, an F1 score of 63.1%, and an AUC of 65.6%. The difference in performance highlights deficiencies of T5 embeddings to distinguish classes effectively, hence requiring consideration of different frameworks for overall quality improvement.

The addition of one-dimensional convolutional neural network (1DCNN) layers greatly enhanced the performance of the T5 model. The BERT + 1DCNN achieved an accuracy rate of 89.5%, which outperformed the 88.8% accuracy rate of the BERT model, and an equally impressive F1-score of 88.7%. Though its recall rate of 89.3% happened to fall marginally lower compared to several other models, it had an AUC of 95.1% and thereby lent indication of an impressive overall performance. Similarly, the T5 + 1DCNN achieved an accuracy of 88.5% and an F1-score of 87.7%, reflecting a level of performance that is overall quite similar to that of the BERT model. The T5 + 1DCNN model achieved an AUC of 95.5%, which demonstrated a small improvement from its BERT counterpart for that specific metric.

The use of dense neural networks, though not outperforming 1D CNN models on all metrics considered, still produced competitive outputs. The BERT + Dense model had an accuracy rate of 87.3%, an AUC of 86.7%, and an F1-score of 93.1%.On the other hand, T5 + Dense attained an accuracy and an AUC of 86.1 and 93.0%, and an F1-score of 85.4. Lastly, our proposed approach sends embeddings from BERT and T5 through two parallel streams, each comprising two branches composed of one using a 1DCNN and another using a dense network before their outputs are combined.

The integration of four model combinations: BERT + 1DCNN, T5 + 1DCNN, BERT + Dense, and T5 + Dense, demonstrated superior performance compared to any single configuration. The combined model achieved an accuracy of 91.3%, an F1-score of 90.0%, and an AUC of 95.6%. Notably, its precision reached 95.2%, indicating a strong ability to reduce false positives and enhance the reliability of detected depressive cases. This emphasis on precision helps minimize unnecessary referrals and ensures that individuals identified as depressed are highly likely to require clinical attention. We acknowledge that the moderate decline in recall (from 89.5 to 86.4%) represents the cost of this gain in precision; however, the overall improvement across other metrics, particularly F1 and AUC, which reflect balanced and discriminative performance.

The confusion matrix and ROC curves of the proposed method are shown in Figure 4, while the accuracy, precision, recall, and F1-score are reported in Table 7. To provide further context on computational feasibility, we report training time and model size for each branch in Table 7. These values quantify the per-epoch cost and the number of trainable parameters. For the ensemble, TM and MS depend on the training strategy: if branches are trained sequentially, the cost is the sum of all branches, whereas in a parallel setup the cost corresponds to the maximum branch. Detailed runtime per epoch and reproducibility logs are available in the accompanying GitHub repository.

**Table 7:** Ablation study.

**Model**	**Acc**.	**Prec**.	**Rec**.	**F1**	**AUC**	**TM (s)**	**MS (M)**
BERT	88.8	86.6	**89.5**	88.0	92.8	1844.8	110
T5	64.1	68.3	64.1	63.1	65.6	4519.0	223
BERT + Dense	87.3	84.4	89.1	86.7	93.1	161.5^a^	116
T5 + Dense	86.1	83.3	87.6	85.4	93.0	161.2^a^	229
BERT + 1DCNN	89.5	88.0	89.3	88.7	95.1	49.0^a^	111
T5 + 1DCNN	88.5	86.6	88.8	87.7	95.5	48.8^a^	223
(Proposed)	**91.3**	**95.2**	86.4	**90.0**	**95.6**	N/A	N/A

The highest value for each model is highlighted in bold.

TM, training time; MS, model size.

^a^Training time of Dense or 1DCNN using extracted embeddings from BERT or T5.

### 5.4 Comparison with the state-of-the-art methods

To provide a more comprehensive assessment of the proposed approaches, we additionally experimented with a Generative Pre-trained Transformer (GPT)-based model and a Temporal Graph model (TGCN), while also replicating several state-of-the-art baselines for direct comparison. Table 8 provides a summary of the evaluation metrics.

**Table 8:** Comparison with the state-of-the-art methods.

**Study**	**Method**	**Accuracy (%)**	**Precision (%)**	**Recall (%)**	**F1 (%)**
Zhang and Guo (2024)	BERT, T5, Dense	89.13	80.0	85.71	82.76
Ours	BERT, T5, Dense	88.2	88.2	**88.4**	88.2
Milintsevich et al. (2023)	S-RoBERTa, BiLSTM	–	–	–	80.6
Ours	S-RoBERTa, BiLSTM	86.1	86.1	86.3	86.1
Villatoro-Tello et al. (2021)	Multi-layer Perceptron	–	87.0	81.0	83.0
Ours	Multi-layer Perceptron	84.9	84.8	84.9	84.8
Rai et al. (2024)	BERT, BiLSTM	–	83.0	83.0	83.0
Ours	BERT, BiLSTM	73.5	72.0	69.9	71.0
Li et al. (2024)	Heterogeneous graph Att.	–	79.0	80.0	79.0
Ours	Heterogeneous graph Att.	77.7	78.4	77.0	77.2
Ours	LLaMA (GPT-based)	86.3	83.1	88.0	85.5
Ours	TGCN + BERT	74.3	71.2	74.6	72.8
Ours	TGCN + T5	77.8	75.5	76.9	76.2
Proposed	BERT, T5, Dense, 1DCNN	**91.3**	**95.2**	85.4	**90.0**

Att.: Attention mechanism.

Compared to previous works, our dual-stream fusion approach demonstrates stronger generalization by effectively combining contextual textual embeddings (from BERT and T5) with convolutional representations that capture local semantic patterns. The GPT-based model (LLaMA) also achieved competitive results (F1 = 85.5%), highlighting the potential of generative large language models for depression detection. The Temporal Graph TGCN variants incorporating BERT and T5 embeddings achieved moderate but consistent performance (F1 = 72.8 and 76.2%, respectively), suggesting that temporal graph reasoning can capture sequential dependencies in dialogue-level or time-series contexts.

When comparing against other state-of-the-art methods, the following two observations can be drawn:

- Transformers dominate: models such as BERT, T5, and S-RoBERTa continue to lead in performance owing to their superior contextual representation and transfer-learning capabilities.
- Hybrid architectures remain strong: many competitive systems combine transformers with BiLSTM, dense, or convolutional layers to exploit both contextual depth and sequential or local feature learning.

## 6 Discussion and conclusion

This study presents a dual-stream transfer learning framework for text-based depression detection, leveraging transformer-based large language models.

Transfer learning, particularly through BERT and T5, offers substantial advantages over non-TL approaches–especially in tasks requiring nuanced interpretation of linguistic patterns. Although models such as RF and Dense perform competitively in the non-TL setting, they are outperformed by TL-based models in terms of recall and F1. This highlights the strength of contextual embeddings in capturing subtle depressive cues.

When T5 embeddings were processed through a 1DCNN architecture, performance improved significantly. The T5 + 1DCNN model achieved an accuracy of 88.5%, an F1 of 87.7%, and an AUC of 95.5%–a substantial increase over standalone T5, which showed weaker performance (accuracy: 64.1%, F1: 63.1%, AUC: 65.6%). These results suggest that convolutional layers effectively complement T5 embeddings by extracting local sequential patterns that improve representation quality.

The integration of BERT and T5 embeddings through parallel Dense and 1DCNN branches led to further performance improvements. The resulting fusion model, which combines all four branches, achieved the best results across all evaluation metrics, including accuracy (91.3%), F1 score (90.0%), and AUC (95.6%). Its high precision (95.2%) indicates strong reliability in minimizing false positive predictions. Although high recall is critical for depression screening, the proposed model balances sensitivity with improved precision to reduce false alarms in decision-support use. It is important to emphasize that the combination of BERT and T5 is not intended to assert theoretical optimality of a specific model pairing. Instead, this design aims to reduce reliance on a single pre-training bias by leveraging heterogeneous semantic encoders. Such an agreement oriented fusion strategy enhances robustness when modeling subtle and implicitly expressed linguistic markers of depression.

Compared to the best-performing non-TL model (RF + TF-IDF, accuracy: 88.3%), the proposed fusion method improved accuracy by 3.0%, demonstrating its superior effectiveness in real-world, low-resource clinical datasets.

In summary, transfer learning with pre-trained LLMs significantly enhances the capability of automated depression detection systems. By combining BERT and T5 within a dual-stream architecture, this study demonstrates a robust approach that outperforms both traditional and deep learning baselines. Importantly, the proposed framework has potential clinical relevance: it may support early-stage depression screening, reduce the burden on clinicians by providing automated pre-assessment tools, and enable scalable integration into telehealth and digital platforms for mental health monitoring.

Looking ahead, future research could explore the generalizability of this method to other mental health conditions, its extension to multimodal settings (e.g., combining text, audio, and video), and its deployment in clinical environments for scalable and early-stage mental health assessment.

## Data Availability

The original contributions presented in the study are included in the article/supplementary material, further inquiries can be directed to the corresponding author.
